# Overexpression of extracellular superoxide dismutase reduces acute radiation induced lung toxicity

**DOI:** 10.1186/1471-2407-5-59

**Published:** 2005-06-10

**Authors:** Zahid N Rabbani, Mitchell S Anscher, Rodney J Folz, Emerald Archer, Hong Huang, Liguang Chen, Maria L Golson, Thaddeus S Samulski, Mark W Dewhirst, Zeljko Vujaskovic

**Affiliations:** 1 Departments of Radiation Oncology, Duke University Medical Center, Durham, North Carolina USA; 2 Department of Medicine, Division of Pulmonary, Allergy and Critical Care Medicine, Duke University Medical Center, Durham, North Carolina, USA

## Abstract

**Background:**

Acute RT-induced damage to the lung is characterized by inflammatory changes, which proceed to the development of fibrotic lesions in the late phase of injury. Ultimately, complete structural ablation will ensue, if the source of inflammatory / fibrogenic mediators and oxidative stress is not removed or attenuated. Therefore, the purpose of this study is to determine whether overexpression of extracellular superoxide dismutase (EC-SOD) in mice ameliorates acute radiation induced injury by inhibiting activation of TGFβ1 and downregulating the Smad 3 arm of its signal transduction pathway.

**Methods:**

Whole thorax radiation (single dose, 15 Gy) was delivered to EC-SOD overexpressing transgenic (XRT-TG) and wild-type (XRT-WT) animals. Mice were sacrificed at 1 day, 1 week, 3, 6, 10 and 14 weeks. Breathing rates, right lung weights, total/differential leukocyte count, activated TGFβ1 and components of its signal transduction pathway (Smad 3 and p-Smad 2/3) were assessed to determine lung injury.

**Results:**

Irradiated wild-type (XRT-WT) animals exhibited time dependent increase in breathing rates and right lung weights, whereas these parameters were significantly less increased (p < 0.05) at 3, 6, 10 and 14 weeks in irradiated transgenic (XRT-TG) mice. An inflammatory response characterized predominantly by macrophage infiltration was pronounced in XRT-WT mice. This acute inflammation was significantly attenuated (p < 0.05) in XRT-TG animals at 1, 3, 6 and 14 weeks. Expression of activated TGFβ1 and components of its signal transduction pathway were significantly reduced (p < 0.05) at later time-points in XRT-TG *vs. *XRT-WT.

**Conclusion:**

This study shows that overexpression of EC-SOD confers protection against RT-induced acute lung injury. EC-SOD appears to work, in part, via an attenuation of the macrophage response and also decreases TGFβ1 activation with a subsequent downregulation of the profibrotic TGFβ pathway.

## Background

Acute radiation (RT) induced lung toxicity and subsequently occurring pulmonary fibrosis are considered to be critical, dose-limiting factors for radiation therapy of thoracic malignancies [1]. Subclinical molecular and cellular events commence during RT therapy, but the clinical features and histological findings may not be revealed for months, or even years after the treatment [2].

In RT-induced lung injury, damage to endothelial and epithelial cells are thought to be the initial steps leading to acute lung toxicity [3]. Tissue damage and repair initiated by irradiation is associated with the production of important biological mediators, such as cytokines [4]. These cytokines perpetuate the inflammatory and fibrogenic processes associated with RT injury [5]. The relative role of cytokine dysregulation versus direct tissue injury from irradiation in the pathogenesis of acute and late toxicities is not well defined. Previous observations and recent advancements in understanding the role of these cytokines implicate transforming growth factor-β1 (TGF-β) as a key component in the development of RT-induced normal tissue injury in multiple organs, including lungs [6].

In addition to cytokine dysregulation, an oxidant/antioxidant imbalance in the lower respiratory tract has been proposed as the mechanism of lung injury in a number of inflammatory lung conditions [7,8]. Ionizing RT is associated with increased production of free radicals [9], which is reflected by the accumulation of oxidatively damaged cellular macromolecules. RT may impair lung cells either directly via generation of reactive oxygen species (ROS) [9] or indirectly via the action on parenchymal and inflammatory cells through biological mediators [4,5]. This process may subordinate the cellular antioxidant defenses and lead to the accumulation of toxic levels of ROS.

Extracellular superoxide dismutase (EC-SOD), one of the subtypes of naturally occurring superoxide dismutases, is the dominant antioxidant enzyme found in a variety of extracellular compartments [10]. These enzymes act by catalyzing the dismutation of the superoxide anion radical to oxygen and hydrogen peroxide. EC-SOD is secreted into the extracellular spaces, and in the lung it is expressed primarily by the alveolar type II pneumocytes [11]. EC-SOD is mainly bound to the extracellular matrix but it is also detectable in plasma [12]. The protective role of this enzyme has been studied in RT, bleomycin or hyperoxia induced oxidative stress lung injury [13-15].

Recently, we have reported that overexpression of EC-SOD in transgenic mice appears to protect against RT-induced chronic injury [13]. In the present study, we analyzed the early events at the molecular and cellular levels to clarify the mechanisms by which overexpression of EC-SOD protects against RT-induced lung damage. We hypothesized that continuous over-production of ROS, during and well after radiotherapy has been completed, is responsible for the pathogenesis of RT-induced lung injury and that continuous overexpression of EC-SOD would ameliorate this injury.

## Methods

### Animals

Transgenic (TG) B6C3 mice that overexpress human EC-SOD (hEc-SOD) in alveolar and airway epithelial cells and wild-type (WT) littermates were utilized for this study. The generation of the TG mice has been described in detail by *Folz et. al. *[15]. These mice are maintained in a B6C3 background. Heterozygous transgenic positive mice are always bred to an F1 (C57BL/6 × C3H) mouse, and the transgenic positive pups are compared to their transgenic negative littermates. Polymerase chain reaction (PCR) analysis of tail DNA was performed to confirm the genotype status. PCR positive and negative animals were used for all these studies. The animals were maintained in cages at room temperature, with a 12 hr light-dark cycle with access to food and water *ad libitum*. The protocol was approved by the Duke University Medical Center Institutional Animal Care and Use Committee.

### Thoracic irradiation

The animals were randomly distributed into four groups; unirradiated, wild-type (Con -WT), EC-SOD overexpressing transgenic (Con -TG), irradiated EC-SOD overexpressing transgenic (XRT-TG) and wild-type mice (XRT-WT). RT was delivered with a single dorsal-ventral field using 4-MV photons, for a dose of 15 Gy to the whole thorax with 0.5 cm bolus material. Mice were sacrificed at six different time points post irradiation (1 day, 1, 3, 6, 10 and 14 wks), along with matched controls.

### Breathing frequency

Functional assessment of lung damage was performed weekly, by measuring breathing frequency, using a whole-body plethysmography chamber (Model RM-80; Columbus Instruments, Columbus, Ohio). Four readings were taken at each time point for every animal, and the mean value was used for analysis.

### Broncho-alveolar lavage fluid

Mice were anesthetized by intraperitoneal injection of pentobarbital (50 μg/g). After the onset of adequate anesthesia, the trachea was exposed and a 20-gauge needle was used to puncture the trachea, which was replaced with a 21-gauge blunt needle. A silk ligature was fastened around the trachea to secure the needle. Bronchoalveolar lavage (BAL) was performed with 1 ml PBS using a 1-ml syringe and repeated once. The collected fluid was immediately processed as follows. BAL fluid (300 μL) was cytocentrifuged and stained with a leukostat stain kit (Fisher Scientific Co., Pittsburgh, Pennsylvania, USA). A minimum of 300 cells were subsequently counted to obtain a differential cell count. Cellular BALF (30–50 μL) was stained with trypan blue, and the total cell count was manually obtained using a Neubauer hemocytometer (Reichert, Buffalo, New York, USA). The remaining BALF was centrifuged at 1,800 *g *for 5 minutes at 4°C, and the supernatant was decanted and centrifuged a second time at 12,000 *g *for 10 minutes at 4°C. The supernatant was collected and saved at -80°C for further analysis, and the pelleted cells were snap frozen in liquid nitrogen and stored at -80°C.

### Wet lung weights

At the time of sacrifice, the right lung was removed, gently blotted, and the wet weight recorded. The right lung was snap frozen in liquid nitrogen and then stored in -80°C freezer.

### Histopathology

The left lungs from mice in each group and at each time point were fixed in situ for microscopy by intratracheal instillation of 4% paraformaldehyde in PBS at 20 cm H_2_O pressure for 10 minutes and subsequently removed from the thorax and immersed in additional fixative for 4–6 hours. These tissues were paraffin-embedded, sectioned, and stained with hematoxylin and eosin. Histopathologic evidence for lung injury was scored on a scale from 0 (normal) to 4 (most severe) as described earlier [16]. Parameters assessed were the degree of vascular congestion, thickening of alveolar wall (edema), hyaline membrane formation in the distal airway, and inflammatory cell accumulation.

### Tissue TGF-β1 activity

Frozen lung tissue was thawed at room temperature, then placed in 1X phosphate buffered saline (PBS) with 0.5 mM EDTA according to weight (10 μL of buffer per 1 mg of tissue). The sample was homegenized by sonication, then spun at 15,000 rpm for 30 minutes at 4°C. The supernatant was used for the sandwich enzyme-linked immunosorbent assay (ELISA) in order to quantify the TGF-β1. To measure the active TGF-β1, anti-TGF-β1 monoclonal antibody and biotinylated anti-TGF-β1 antibodies were used as the capture and probe antibodies, respectively. Horseradish peroxidase-conjugated with streptavidin was used to bind the biotin, followed by substrate reagent and stop solution (all products are from R&D Systems Inc, Minneapolis, MN). To quantify the total TGF-β1, latent TGF-β1 was activated by adding 0.1 ml 2.5 N Acid / 10 M Urea into 0.1 ml sample and neutralized by adding 0.1 ml 2.7 N NaOH / 1 M Hepes.

Ninety-six-well microtiter plates were read at OD 450 nm using a kinetic microplate reader (v-max; Molecular Devices, Menlo Park, CA). The level of TGF-β1 (total and active) was determined by comparing the peroxidase activity to known concentrations of purified TGF-β1 (R&D Systems Inc, Minneapolis, MN).

### Immunohistochemistry (*Smad3 And p-Smad2/3*)

Immunohistochemistry was carried out as previously described [17]. Briefly, paraffin embedded tissues were sectioned at 5 μm thickness, and deparaffinized and hydrated using a xylene solution and graded ethanol. An endogenous peroxide blocking solution of 3% hydrogen peroxide was applied for 20 minutes at room temperature then rinsed in deionized water. Sections were then placed in an antigen retrieval solution of citrate buffer from Biogenex (San Ramon, CA). The solution was heated in a microwave for 3–4 minutes, cooled for 2–3 minutes, then heated again in a microwave. After cooling at room temperature for 20 minutes, sections were rinsed in deionized water. Next, the sections were incubated in 10% donkey serum in phosphate-buffered saline (PBS) for 25 minutes at room temperature to reduce non-specific binding of antibody. Sections were then rinsed three times with 1X PBS, covered with primary antibodies against the Smad 3 and p-Smad 2/3 (dilution 1:200, Santa Cruz Biotechnology), incubated in a humidity chamber at 37°C for 60 minutes. After rinsing three times with 1% PBS, secondary and tertiary antibodies were added to the sections per manufacturer instructions (314KLD, Innovex, Richmond, CA), with incubation in the humidity chamber at 37°C for 30 minutes. Sections were again rinsed three times in 1% PBS, then incubated for 2 minutes at room temperature in a peroxidase substrate solution with 3, 3'-diaminobenzidine tetrahydrochloride (Sigma, St. Louis, MO). The slides were washed three times for 10 minutes in deionized water, and counterstained with hematoxylin. After rinsing again with deionized water, sections were dehydrated in graded ethanol and xylene, then mounted. Normal serum was used as controls with each set of stains.

### Scoring of positive cells

The lung specimens were subjected to blinded evaluation. After scanning the whole lung section and calculating the extent of damaged lung parenchyma, 4 representative fields (40X objective) were selected and analyzed. After initial qualitative assessment of morphologic changes, cells that showed staining after RT exposure to the Smad-3 and p-Smad-2/3 antibodies were recorded. The sum of the measurement from the 4 fields was totaled, and the arithmetic mean was calculated. Positive cell counts were expressed as the average number of cells per field. Means from lungs of irradiated animals were compared with the corresponding values obtained from control animals.

### Statistical methods

The Student's *t *test and One-way ANOVA was used to test the significance of any differences between groups at each time. A p-value ≤ 0.05 was considered statistically significant. All datas are presented as mean values (± 95% confidence interval).

## Results

### Breathing frequency

**Figure 1:**
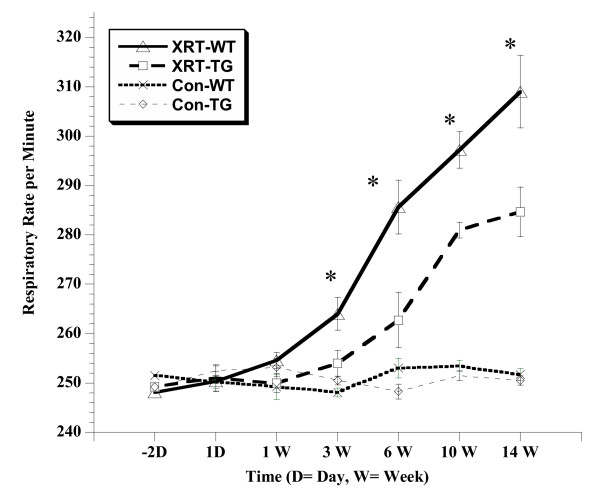
***Comparison of breathing frequency over time***. Changes in breathing rate after 15 Gy of single dose of irradiation to whole thorax in XRT-WT and XRT-TG groups. XRT-WT group showed a significant increase in respiratory rate *vs*. XRT-TG, at 3, 6, 10 and 14 wks, * *p *< 0.05. Error bars represent 95% confidence intervals.

The pulmonary function of the animals was assessed by monitoring the respiratory rate every week for 14 weeks. The XRT-WT mice exhibited a progressive increase in breathing rates after irradiation beginning at 3 weeks in comparison to control animals (Figure 1). XRT-TG animals have a delay in the onset of the functional lung damage (3 weeks), with the significant decrease (p < 0.05) in respiratory rate as compared to XRT-WT mice. During the follow-up period of 14 weeks the mean breathing rates of the two control groups were not changed from baseline values (249 ± 2.5 *vs. *251 ± 2).

### Right lung wet weights

**Figure 2:**
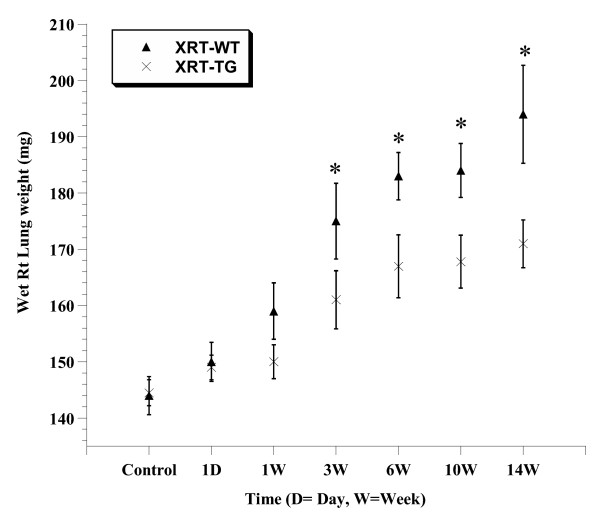
***Comparison of right lung wet weights over time***. Lung weights at 1 day, 1,3, 6, 10 and 14 weeks of exposure to radiation. A significant increase in the wet weight of XRT-WT lungs was observed by 3 wks of radiation exposure (XRT-WT *vs*. XRT-TG, at 3, 6, 10 and14 wks, * *p *< 0.05). Error bars represent 95% confidence intervals.

The amount of acute lung damage elicited by RT was measured by an increase in right lung wet weights, which are an index of pulmonary edema and consolidation. RT treatment resulted in a significant increase in right lung wet weights in the XRT-WT group (Figure 2). The XRT-TG mouse strain showed significantly less (p < 0.05) increase in wet weight from 3 week to 14 weeks, when compared with their XRT-WT littermates.

### Broncho-alveolar lavage fluid cell counts

**Figure 3:**
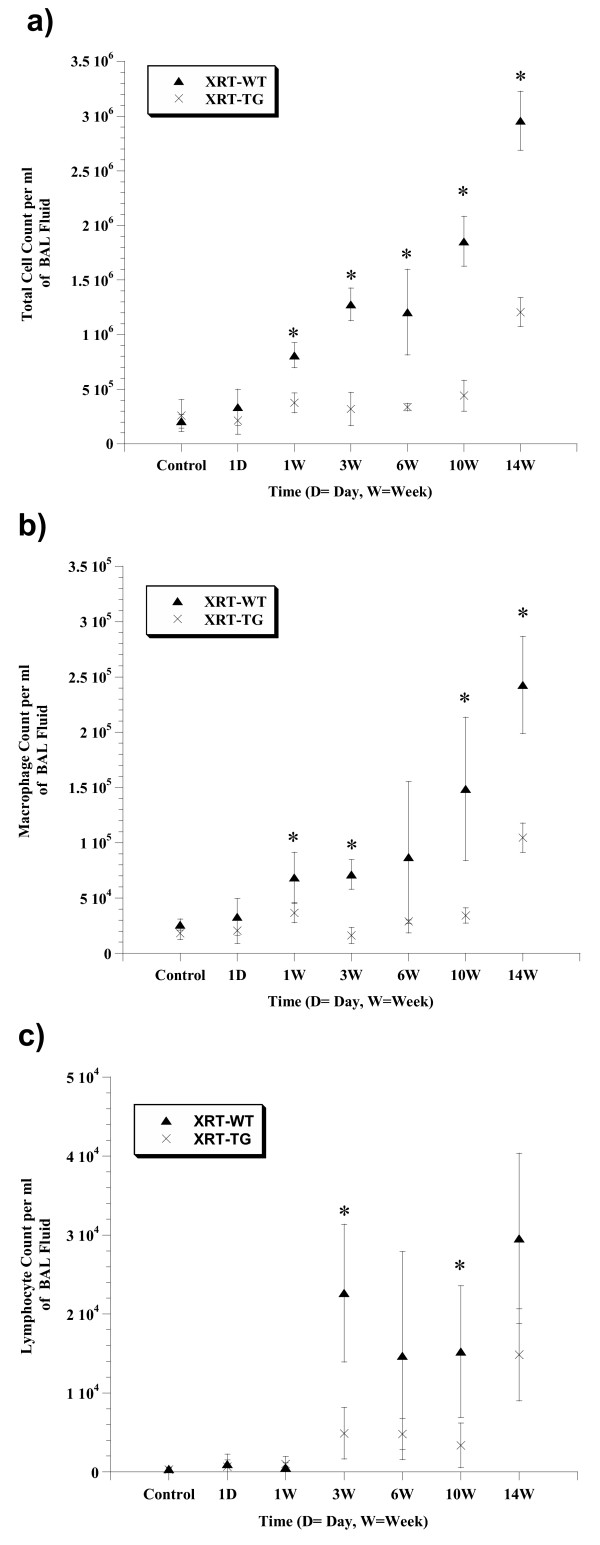
*Comparison of cell counts in bronchoalveolar lavage fluid (BALF) *from XRT-WT and XRT-TG mice exposed to radiation. (*a*) Total cell counts. and (*b*) Macrophage cell counts at 1day, 1,3, 6, 10 and 14 weeks of exposure to radiation. Both total cell and macrophage counts significantly increased (* *p *< 0.05) with time of exposure in XRT-WT animals (*c*) Lymphocyte cells count was significantly increased by 3 wk, in the BALF of the XRT-WT group as compared to the XRT-TG mice (XRT-WT *vs*. XRT-TG, at 3, and 10 wks, * *p *< 0.05). Error bars represent 95% confidence intervals.

There was no difference in total cell counts between XRT-WT and XRT-TG mice 1 day after irradiation (Figure 3a). However, the total cell count increased significantly by 1 week in the XRT-WT mice when compared with the XRT-TG mice and remained elevated till 14 weeks (p < 0.05).

The increase in BALF cell count was mainly due to an increase in the number of macrophages (Figure 3b). Beginning at 1 week, the XRT-WT mice showed a significantly greater increase in macrophages, which persisted throughout the period of observation (XRT-WT *vs*. XRT-TG, at 1, 3, 10, and 14 weeks, p < 0.05).

The lymphocyte count was also significantly increased in XRT-WT animals beginning at 3 weeks (Figure 3c) as compared to the XRT-TG animals (XRT-WT *vs*. XRT-TG, at 3 and 10 weeks, p < 0.05).

### Histopathology

**Figure 4:**
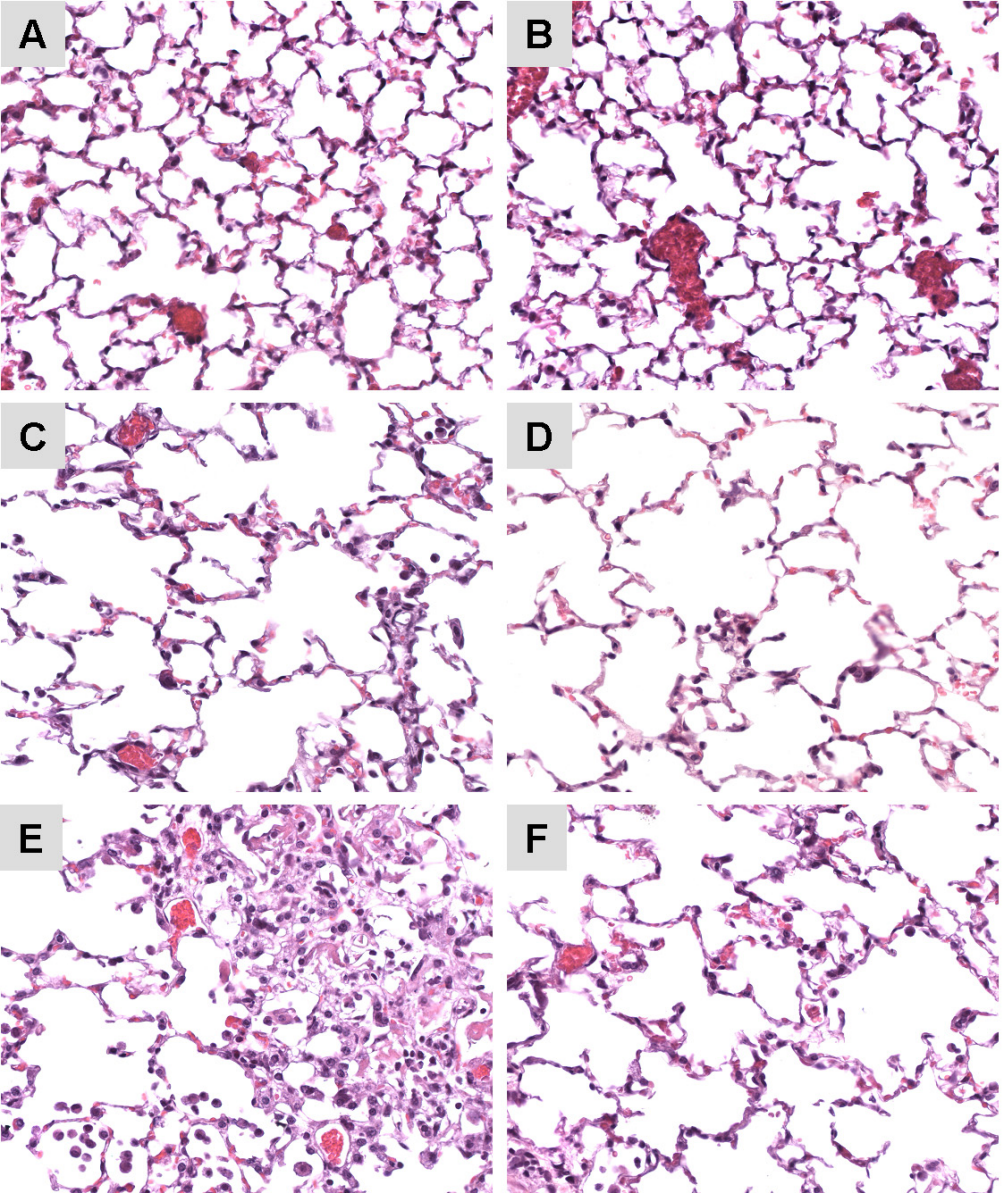
*Light microscopy of hematoxylin and eosin-stained section *of representative lungs from controls (***A, B ***) and treatment groups (XRT-WT *vs*. XRT-TG) at 3 wk (***C, D ***) and 14 wks (***E, F ***). Representative lung sections from XRT-WT exposed to 3 wk of radiation showing mild to moderate damage at 3 wk after radiation (***C ***), which gradually increased with time course of injury and become moderate to severe with increased thickening of alveolar wall and enhanced infiltration composed mainly of macrophages and other inflammatory cells at 14 weeks (***E ***). Whereas the XRT-TG mice showing mild-modest alveolar septal thickness and fewer numbers of alveolar macrophages and other inflammatory cells (***D, F ***). Magnification × 400.

At 3 weeks after irradiation, the H & E stained lung sections of XRT-WT mice showed evidence for decreased pulmonary compliance, as indicated by increased edema, thickening of the alveolar walls, vascular and interstitial congestion, and diffuse inflammatory cell infiltration, which worsened with time (Figure. 4C, E). Histological changes were also observed in XRT-TG mice after 3 weeks (Figure. 4D); however they were lesser in degree and sparse in distribution compared to the XRT-WT animals (Figure. 4C). In the later time points, the extent and severity of injury was greater in XRT-WT groups compared to their XRT-TG animals (Figure 4E, F).

### TGFβ activity

**Figure 5:**
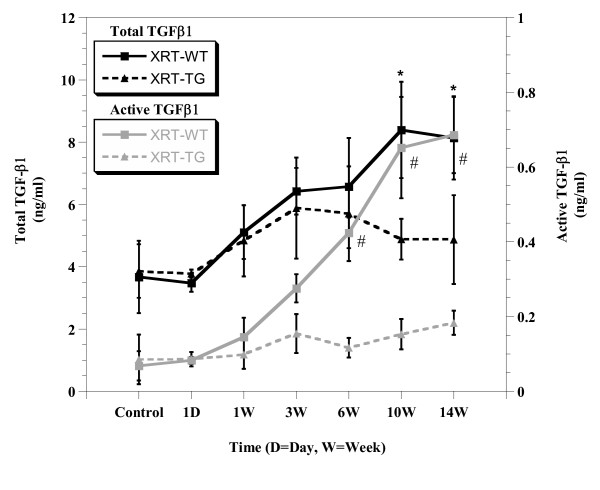
***Comparison of TGFβ1 activation in irradiated lung tissue***. Increase active TGFβ1 expression began by 1 week. By 6 weeks, there was significantly more activated TGFβ1 in the lungs of XRT-WT mice (grey squares) vs. XRT-TG animals (grey triangles) (XRT-WT *vs*. XRT-TG, at 6, 10 and 14 wks, ^# ^*p *< 0.05). There were significantly more total TGFβ1 levels in XRT-WT (black squares) vs. XRT-TG animals (black triangles) at late time points (XRT-WT *vs*. XRT-TG, at 10 and 14 wks, * *p *< 0.05). Error bars represent 95% confidence intervals.

The results of the quantitative assessment of levels of active and total TGFβ1 protein expression in lung tissue are shown in Figure 5. Unirradiated lung tissue of control animals expressed lower levels of TGFβ1. Following thoracic irradiation, lung tissue levels of active TGFβ1 in XRT-WT were increased within the first week. The active TGFβ1 levels were significantly increased from 6 weeks onward. The XRT-TG animals demonstrated significantly less TGFβ1 activation as compared to XRT-WT mice (XRT-WT *vs*. XRT-TG, at 6, 10, and 14 weeks, p < 0.05, Figure 5).

### Signal transduction

**Figure 6:**
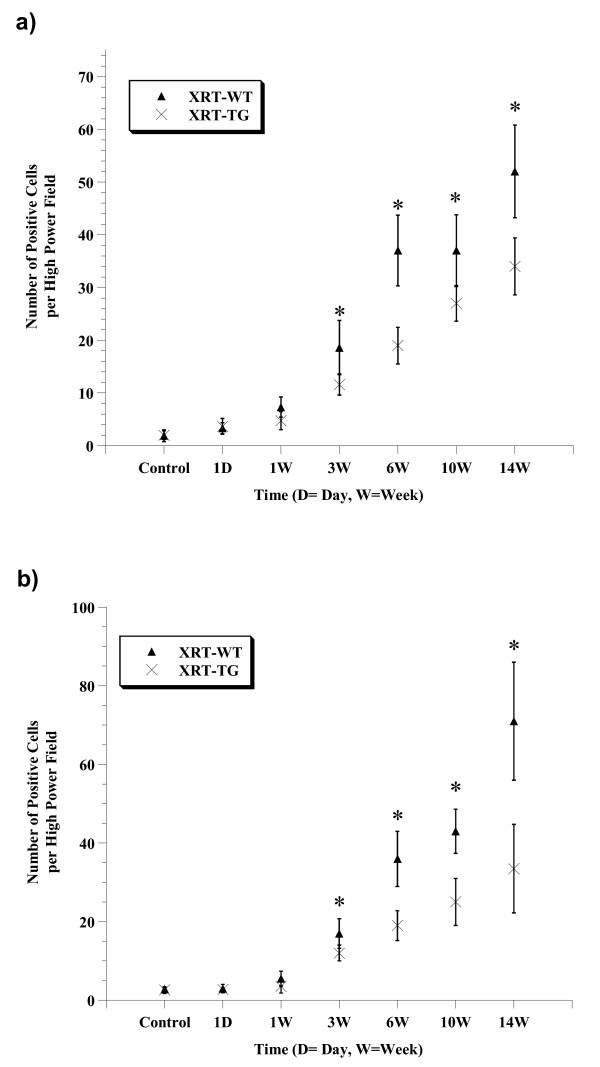
**a): *Smad3 expression after irradiation. ***Cells expressing Smad3 were counted at 40X magnification in four random fields by two blinded observers. The Smad3 positive cell count for each animal was the mean of all eight readings. Increased expression of Smad3 in XRT-WT lungs was noticeable at 1 wk, and which steadily increased from 3–14 wks. This temporal increase in XRT-TG was significantly reduced than XRT-WT (XRT-WT *vs*. XRT-TG, at 3, 6, 10 and14 wks, * *p *< 0.05). **b): p-Smad2/3 expression after irradiation**. For p-Smad2/3, cell positivity was counted at 40X and mean value for each animal was taken. In XRT-WT lung, there was significant increase in p-Smad2/3 expression by 3 weeks post irradiation. With time, the fraction of positively staining cells also increased in XRT-TG animals but the level of expression in this group was significantly decreased when compared with XRT-WT (XRT-WT *vs*. XRT-TG, at 3, 6, 10 and 14 wks, * *p *< 0.05). Error bars represent 95% confidence intervals.

**Figure 7:**
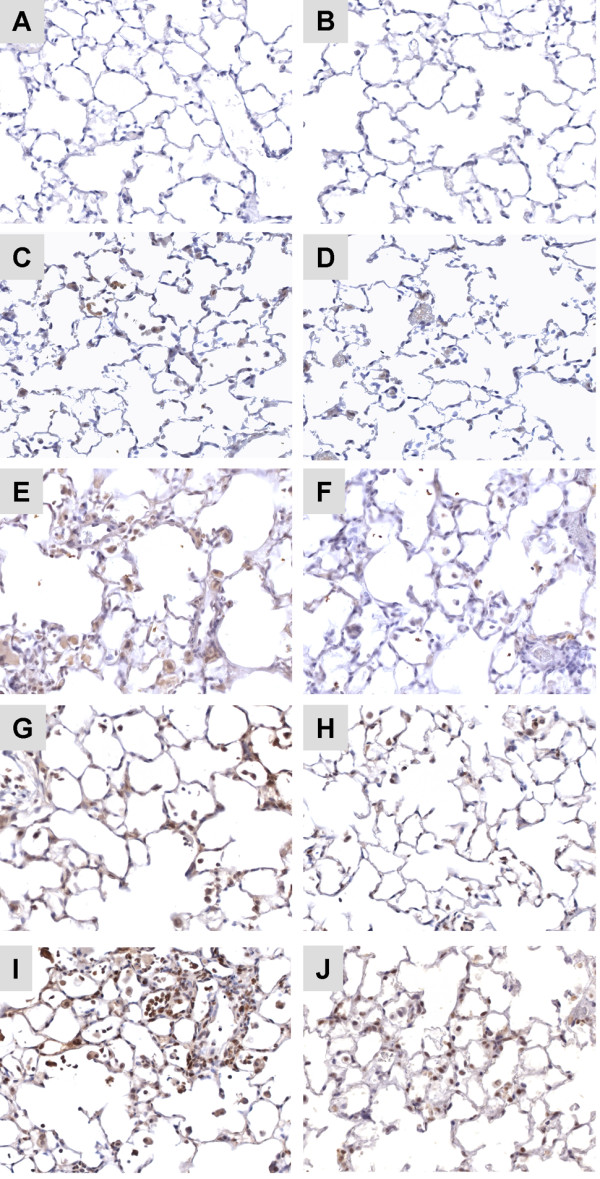
*Representative images of Smad3 immunohistochemistry *of controls (***A, B ***) and XRT-WT *vs*. XRT-TG groups at 3 wk (***C, D ***) and 14 wks (***E, F ***). For Smad3, most positively stained cells are present in damaged areas of XRT-WT lungs, which showed significantly increased cell positivity when compared with XRT-TG animals. Representative images of p-Smad2/3 immunohistochemistry of XRT-WT *vs*. XRT-TG groups at 3 wk (***G, H ***) and 14 wks (***I, J ***) after irradiation. XRT-WT animals exhibited significantly increased positivity for p-Smad2/3 when compared with XRT-TG mice. See the results section for the details. Magnification × 400.

Increased immunoreactivity for Smad 3 in lung parenchyma of XRT-WT animals was noticeable at 1 week, and which steadily increased from 3–14 weeks. The temporal increase with XRT-TG was less steep than XRT-WT (XRT-WT *vs*. XRT-TG, at 3, 6, 10 and 14 weeks, p < 0.05, Figure 6a) (Representative images of XRT-WT vs. XRT-TG at 3 and 14 weeks after irradiation shown in Figure 7C–F).

In XRT-WT mice, there was significant increase in p-Smad2/3 positivity by 3 weeks post irradiation (XRT-WT *vs*. XRT-TG, at 3, 6, 10 and 14 weeks, p < 0.05, Figure 6b). With time, the fraction of positively staining cells also increased in XRT-TG animals but this p-Smad2/3 expression was significantly diminished when compared with XRT-WT. Thus, RT not only increased TGFβ expression and activation, but also increased signal transduction down the fibrosis pathway. This effect was significantly attenuated by over-expression of ECSOD. (Representative images of XRT-WT vs. XRT-TG at 3 and 14 weeks after irradiation shown in Figure 7G–J).

## Discussion

The current evidence indicates that acute RT-induced damage to the lung is characterized by inflammatory changes, which proceed to the development of fibrotic lesions in the late phase. Ultimately, complete structural ablation will ensue, if the source of inflammatory/fibrogenic mediators and oxidative stress is not removed or attenuated [13]. Our data demonstrate that overexpression of ECSOD in mouse lung appears to confer protection from acute RT damage by reducing the inflammatory response, decreasing in TGFβ1 activation and, consequently, downregulation of TGFβ signal transduction pathway.

Prior work by our laboratory has revealed that EC-SOD is protective against chronic RT-induced lung injury, but we were unable to differentiate between the acute phase inflammatory response (pneumonitis /alveolitis) and the fibrotic response to pulmonary irradiation because only a single late time point was studied [13]. To begin to answer this question, we used TG mice in which alveolar and airway epithelial cells overexpress EC-SOD. Thus, by using this TG mouse model we were able to enhance the antioxidant capacity of the extracellular pulmonary compartment in which antioxidant reserves may play critical roles in preserving pulmonary function against early RT damage.

The lung is among one of the most radiosensitive viscera to ionizing RT. Ionizing RT has long been recognized to induce an inflammatory response within irradiated tissues [18-22]. Strong evidence implicates a key role for inflammation in the development of radiation induced pulmonary injury [23]. Suppression of this acute inflammation may lead to a reduction in subsequent fibrosis.

The mechanism underlying the inflammatory response in RT-induced lung injury is complex. Endothelial damage leads to leukocyte adhesion and transmigration into tissues. Inflammatory cells are a known source of ROS [24,25]. These cells can also release inflammatory and fibrogenic cytokines [26], which further contribute to injury in part through the recruitment of more inflammatory cells to the site of injury [5]. The time course for recruitment of the inflammatory cells in the lower respiratory tract of XRT-WT animals in this study demonstrates the important role of inflammation in early radiation injury. In the presence of a potent anti-inflammatory stimulus, i.e., EC-SOD, XRT-TG animals suffered significantly less RT-induced pulmonary damage.

The anti-inflammatory effects of EC-SOD also appear to reduce the risk of subsequent fibrosis. The elevated levels of EC-SOD in transgenic mice has been shown to be protective against the development of pulmonary fibrosis from a number of stimuli, including RT, hyperoxia and bleomycin induced pulmonary fibrosis [13-15]. EC-SOD TG mice have been shown to better tolerate hyperoxia, in part by reducing the excessive influx of inflammatory cells into the lung [15]. In a mouse model of influenza virus-induced pneumonia, the overexpression of EC-SOD markedly ameliorated the inflammatory and oxidant responses in the lung [27]. This protective effect of EC-SOD was attributed to inhibitory action against a series of inflammatory mediators. Similar effects were also noticed after exogenous administration of SOD's [28,29] in influenza- induced lung injury.

In contrast to EC-SOD overexpressing animals, EC-SOD knockout mice have shown evidence for increased sensitivity to hyperoxia exposure, which was exhibited by earlier onset of inflammatory reactions and interstitial edema, when compared with wild-type mice [30]. In another study, EC-SOD null mice demonstrated increased susceptibility to inflammation and pulmonary fibrosis. From these results the authors proposed that one mechanism by which EC-SOD protects against pulmonary fibrosis is by inhibiting inflammation [31]. This appears to be the case following radiation as well.

One of the means by which EC-SOD appears to reduce pulmonary inflammation is through suppressing activation and recruitment of macrophages. Macrophages increase in number shortly after irradiation and have been shown to produce a broad spectrum of cytokines, which stimulate fibroblast proliferation and the production of an extracellular matrix [32]. These include platelet-derived growth factor (PDGF) [33], interleukin-1 (IL-1) [34], tumor necrosis factor α (TNFα) [35], basic fibroblast growth factor (bFGF) [36], and TGFβ [37]. These cytokines not only regulate fibroblast function and collagen synthesis, but also recruit other inflammatory cells and contribute to the pathogenesis of both acute and late RT damage [38]. The macrophage count of XRT-TG mice in this study at different time points revealed a significant reduction when compared with XRT-WT. This observation supports the notion that the protective effect of EC-SOD is mediated, at least in part, by its ability to attenuate the influx of macrophages.

Several lines of evidence have supported the use of therapeutic strategies targeting TGFβ1 to prevent fibroproliferative disorders of various organs [3]. Due to its implication in lung fibrogenesis, blockade of the TGFβ pathway has been proposed as a molecular strategy to ameliorate pulmonary fibrosis and is therefore of potential clinical significance [39]. Using a soluble version of TGFβ type II receptor, Wang *et al *[40] found that pulmonary fibrosis, including excess collagen accumulation, was greatly reduced in hamsters after bleomycin administration. Similarly, we found that soluble TGFβ type II receptor gene therapy led to a significant reduction of acute RT-induced lung injury in rats 4 weeks after irradiation [41]. Interference with the TGFβ signal transduction pathway has also been shown to protect against fibrosis formation. Recently, Smad7, an intracellular antagonist of TGFβ signaling, has been shown to attenuate bleomycin-induced lung fibrosis in mice receiving intratracheal injection of recombinant adenovirus overexpressing Smad7 [42]. Smad3 knockout mice have been shown to be protected from RT-induced soft tissue injury [43]. This loss of Smad3 interfered with the chemotactic response of neutrophils, macrophages, keratinocytes, and fibroblasts to TGFβ [44-47]. Upregulation of TGFβ at sites of injury was also Smad3 dependent [43].

Our data further support the notion that TGFβ1 signaling through the Smad 3 pathway is critical to the development of RT-induced injury. Overexpression of EC-SOD leads to downregulation of this signaling pathway as evidenced by both reduced expression and phosphorylation of Smad3. Inhibition of Smad3, or even partial reduction in its activity might afford protection against the toxicities of irradiation. Taken together, these results suggest that abrogation of TGFβ signaling might be sufficient to offer protection against lung fibrogenesis.

In summary, this study shows that overexpression of EC-SOD confers protection against RT induced acute lung injury. EC-SOD appears to work, in part, via an attenuation of the macrophage response. In addition, sustained EC-SOD expression decreases TGFβ activation with a subsequent downregulation of the profibrotic TGF-β pathway. Thus, EC-SOD in the extracellular compartments of the lung is an important modulator of the complex response of normal tissue to acute radiation insult.

## Abbreviations

**TGFβ1 **: Transforming growth factor Beta

**EC-SOD **: Extracellular Superoxide Dismutase

**RT **: Radiation

**XRT-WT **: Irradiated wildtype

**XRT-TG **: Irradiated transgenic

**ROS **: Reactive oxygen species

**BALF **: Broncho-alveolar lavage fluid

## Competing interests

The author(s) declare that they have no competing interests.

## Authors' contributions

**ZNR **performed the study (breathing frequency, wet lung weight, IHC, statistical analysis of the data) and drafted the manuscript. **RJF **and **MG **provided animals and assisted in BALF cell count analysis. **EA **and **LC **helped in histology/IHC and assisted in conducting the study. **TVS **performed whole thorax irradiation to the animals. HH performed TGF-β ELISA. **MWD **helped in editing of manuscript. **ZV **and **MSA **conceived of the study, participated in its design and coordination, and assisted in editing of manuscript. All authors read and approved the final manuscript.

## Pre-publication history

The pre-publication history for this paper can be accessed here:
